# The Role of Bacteria–Mitochondria Communication in the Activation of Neuronal Innate Immunity: Implications to Parkinson’s Disease

**DOI:** 10.3390/ijms24054339

**Published:** 2023-02-22

**Authors:** João D. Magalhães, Ana Raquel Esteves, Emanuel Candeias, Diana F. Silva, Nuno Empadinhas, Sandra Morais Cardoso

**Affiliations:** 1 CNC—Center for Neuroscience and Cell Biology and CIBB—Center for Innovative Biomedicine and Biotechnology, University of Coimbra, 3004-504 Coimbra, Portugal; 2 Ph.D. Programme in Biomedicine and Experimental Biology (PDBEB), Institute for Interdisciplinary Research, University of Coimbra, 3004-504 Coimbra, Portugal; 3 Institute of Cellular and Molecular Biology, Faculty of Medicine, University of Coimbra, 3000-548 Coimbra, Portugal

**Keywords:** mitochondria, alphaproteobacteria, innate immunity, antimicrobial peptides, α-Synuclein

## Abstract

Mitochondria play a key role in regulating host metabolism, immunity and cellular homeostasis. Remarkably, these organelles are proposed to have evolved from an endosymbiotic association between an alphaproteobacterium and a primitive eukaryotic host cell or an archaeon. This crucial event determined that human cell mitochondria share some features with bacteria, namely cardiolipin, N-formyl peptides, mtDNA and transcription factor A, that can act as mitochondrial-derived damage-associated molecular patterns (DAMPs). The impact of extracellular bacteria on the host act largely through the modulation of mitochondrial activities, and often mitochondria are themselves immunogenic organelles that can trigger protective mechanisms through DAMPs mobilization. In this work, we demonstrate that mesencephalic neurons exposed to an environmental alphaproteobacterium activate innate immunity through toll-like receptor 4 and Nod-like receptor 3. Moreover, we show that mesencephalic neurons increase the expression and aggregation of alpha-synuclein that interacts with mitochondria, leading to their dysfunction. Mitochondrial dynamic alterations also affect mitophagy which favors a positive feedback loop on innate immunity signaling. Our results help to elucidate how bacteria and neuronal mitochondria interact and trigger neuronal damage and neuroinflammation and allow us to discuss the role of bacterial-derived pathogen-associated molecular patterns (PAMPs) in Parkinson’s disease etiology.

## 1. Introduction

Parkinson’s disease (PD) is the second most common neurodegenerative disorder, characterized by the progressive loss of movement control, gaiting and bradykinesia, in consequence of substantia nigra pars compacta (SNpc) neuronal degeneration [1]. The pathophysiological traits of the disease are the presence of Lewy bodies (LBs) in the brain, mainly composed by aggregated alpha-synuclein (α-Syn) [2], microgliosis [3,4] and mitochondrial dysfunction [5]. The neuroinflammatory hypothesis for PD is based on pathological findings showing an increased expression of pro-inflammatory mediators in affected brain areas, DNA polymorphisms of different pro-inflammatory cytokine genes that modify the risk of PD, and finally, epidemiological studies demonstrating that nonsteroidal anti-inflammatory drugs users have a lower risk of developing PD [6]. Additionally, mitochondria dysfunction also plays a key role in the etiology of sporadic PD [5], acting as a hub that connects neuroinflammation and α-Syn expression and aggregation [7]. Indeed, LBs often harbor mitochondrial components [8], which corroborates the fact that neuronal mitochondria are severely affected in PD patients’ brains. Post-mortem studies in PD patients identified alterations in the mitochondrial electron transport chain (ETC), highlighted by a decrease in complexes I and III activities [9,10]. Moreover, several PD mouse models induced with mitochondrial toxins recapitulate some PD features [11,12,13]. Mitochondrial dysfunction induced by rotenone is sufficient to promote the accumulation of p-S129 α-Syn, the pathological form of α-Syn, in mice brains [14]. Studies in post-mortem brains of PD patients revealed that neuronal mitochondria appear to be smaller and swelled [15], suggesting a more reticulated mitochondrial network than age-matched controls. In fact, it was observed that treatment with MPTP, a toxin that targets mitochondria, induced phosphorylation of DRP1, resulting in mitochondrial fragmentation [16]. Moreover, the proteins responsible for mitochondrial elongation, mitofusins 1 and 2, were found to be recruited and sent for degradation by the PINK/Parkin axis in homeostatic conditions [17,18] to allow mitophagy.

Mitochondria are distinctive organelles that possess their own DNA (mtDNA) and replication processes independent of their host cellular division. Furthermore, mitochondria harbor unique ribosomes and contain cardiolipin (named after its initial identification in animal hearts) in the inner membrane, a diphosphatidylglycerol lipid also found in the membrane of most bacteria [19,20,21]. This may be explained in light of the endosymbiotic theory, where mitochondria are proposed to have evolved from ancestor Proteobacteria that were engulfed by an archaeal or a different proto-eukaryotic host [22,23] but conserved some of their characteristics during evolution. Since their components are distinct from the rest of the mammalian cellular elements, they are recognized as damage-associated molecular patterns (DAMPs) by toll-like receptors (TLRs) and Nod-like receptors (NLRs) when they are released to the surrounding cytosol [24,25]. In fact, several TLRs are crucial players in disease modulation in PD. A study revealed that neuronal TLR2 is specifically upregulated in the anterior cingulate cortex and substantia nigra in PD [26]. It was also observed that TLR4 expression is essential for the pathogenesis of PD [27,28]. In a mouse model of PD, it was found that neuronal TLR4 ablation was protective [29], halting inflammasome formation and consequent dopaminergic degeneration, thus corroborating the role of TLR4 in PD.

In PD neurons, the mitochondrial network is fragmented, which leads to prolonged exposure to mitochondrial DAMPs, triggering a chronic inflammatory response denominated as “sterile inflammation” [30]. Indeed, mitochondrial DAMPs may be released from injured cells and signal other cells [31]. For instance, in our previous studies, we verified that a bacterial metabolite induced neuronal immune activation by targeting mitochondria [11,32]. An increased release of cytochrome c in a cellular model of PD using rotenone was also observed [33]. Cytochrome c can also be recognized by TLR4 and elicit an inflammatory response [34].

Innate immunity activation generates a multitude of cellular responses. TLR and NLR activation not only trigger the inflammatory cascade, but also induce the expression of antimicrobial peptides, a crucial response against bacterial infections. For instance, it was observed that TLR2 is essential to drive the expression of antimicrobial peptide human β-defensin 2 [35]. Likewise, the activation of the heterodimer TLR2/1 is essential in monocytes to activate the production of antimicrobial peptide β-defensin 4 in response to Mycobacterium tuberculosis infection [36]. Notably, the ablation of TLR4 prevents the deleterious effects of rotenone, a complex I inhibitor [37]. Interestingly, α-Syn may also be recognized as a DAMP by astrocytes due to its interaction with TLR4 [38]. Since the innate immune response is extremely well conserved, it is reasonable to believe that neuronal α-Syn- and mitochondrial-released DAMPs can induce autocrine or paracrine signaling pathways [39,40].

Herein we show that an environmental proteobacterium strain can activate innate immunity in mesencephalic neurons, partly through the activation of TLR4 and NLRP3 signaling pathways. α-Syn is expressed upon exposure of neurons to the bacteria and aggregates inside mitochondria, whose dysfunction leads to the fragmentation of their network to promote their degradation by mitophagy. Although we observed activation of the autophagic pathway, we also detected a decrease in the autophagic flow, which potentiated the accumulation of defective mitochondria, thus contributing to the exposure of additional DAMPs and further activation of innate immunity in a self-amplified cycle with obvious deleterious effects.

## 2. Results

### 2.1. Bacteria-Mediated Neuronal Innate Immunity Activation: The Role of α-Syn

**Figure 1:**
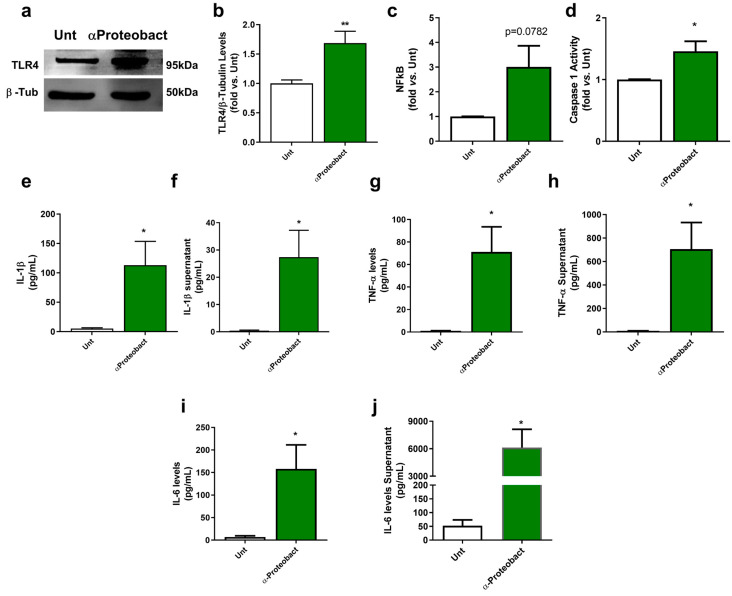
Bacterial exposure activates neuronal innate immunity. Primary mesencephalic neuronal cultures from naive mice were exposed to 1 × 10^6^/mL bacteria for 48 hours (**a**) Representative immunoblot for TLR4. Blots were re-probed for β-Tubulin to confirm equal protein loading. (**b**) Densitometric analysis of the levels of TLR4 was normalized with β-Tubulin. Data are expressed relatively to untreated (Unt) neurons (*n* = 7, ** *p* = 0.0065). (**c**) Nuclear factor kappa-B (NF-κB) levels were calculated using NF-κB p65 ELISA kit. Data are expressed relatively to Unt neurons (*n* = 3, *p* = 0.0782). (**d**) Caspase-1 activation. Data are expressed relatively to Unt neurons (*n* = 3, * *p* = 0.0459). (**e**) IL-1β levels in the isolated cytosolic fraction was determined using an IL-1β ELISA kit. Values are pg/mL (*n* = 5–6, * *p* = 0.0399). (**f**) IL-1β levels released by neurons were determined using an IL-1β ELISA kit. Values are pg/mL (*n* = 4–5, * *p* = 0.0459). (**g**) TNFα levels in the isolated cytosolic fraction were determined using a TNFα ELISA kit. Values are pg/mL (*n* = 3, * *p* = 0.0353). (**h**) TNFα levels released by neurons were determined using a TNFα ELISA kit. Values are pg/mL (*n* = 3, * *p* = 0.0372). (**i**) IL-6 levels in the isolated cytosolic fraction were determined using an IL-6 ELISA kit. Values are pg/mL (*n* = 4, * *p* = 0.0301). (**j**) IL-6 levels released by neurons were determined using an IL-6 ELISA kit. Values are pg/mL (*n* = 4, * *p* = 0.0226). Data represent mean + SEM. Unpaired Student’s *t*-test was performed.

Bacteria invading the gut mucosa or the brain parenchyma can infect multiple neural cell types, leading to inflammation with dysfunction of neural networks and excitotoxicity, regional damage and cell death [41,42]. Indeed, Proteobacteria are gram-negative bacteria that expose lipopolysaccharides (LPS) in their outer membrane and activate innate immunity through TLR4 [43]. To guarantee that we are tackling neuronal contribution to innate immunity activation, we treated primary neuronal cultures with FDU [44] to keep a low level of glial cell contamination in primary mesencephalic neuronal cultures (less than 1% of Iba1, CD11b+ or Trem2-positive cells and less than 20% of GFAP+ cells) [11]. Our data show that neurons exposed to the bacteria increased TLR4 levels (Figure 1a,b; *n* = 7). TLR4 can activate NF-κB signaling pathway and regulate pro-inflammatory cytokine expression. We observed that bacteria led to the activation of NF-kB, although at the time point selected for the study, we do not see statistical significance (Figure 1c; *n* = 3). However, we observed caspase-1 activation (Figure 1d; *n* = 3) associated with NLRP3 inflammasome, which promotes pro-IL-1β cleavage into its mature form (Figure 1e; *n* = 5–6) to be released (Figure 1f; *n* = 4–5). Indeed, NF-κB is required for the induction of a large number of inflammatory genes [45], including those encoding IL-1β, TNF-α and IL-6. Additionally, we observed that these neurons also produce and release other inflammatory cytokines, such as TNF-α (Figure 1g,h; *n* = 3) and IL-6 (Figure 1i,j; *n* = 4), which may mediate innate immunity in the absence of glial cells.

**Figure 2:**
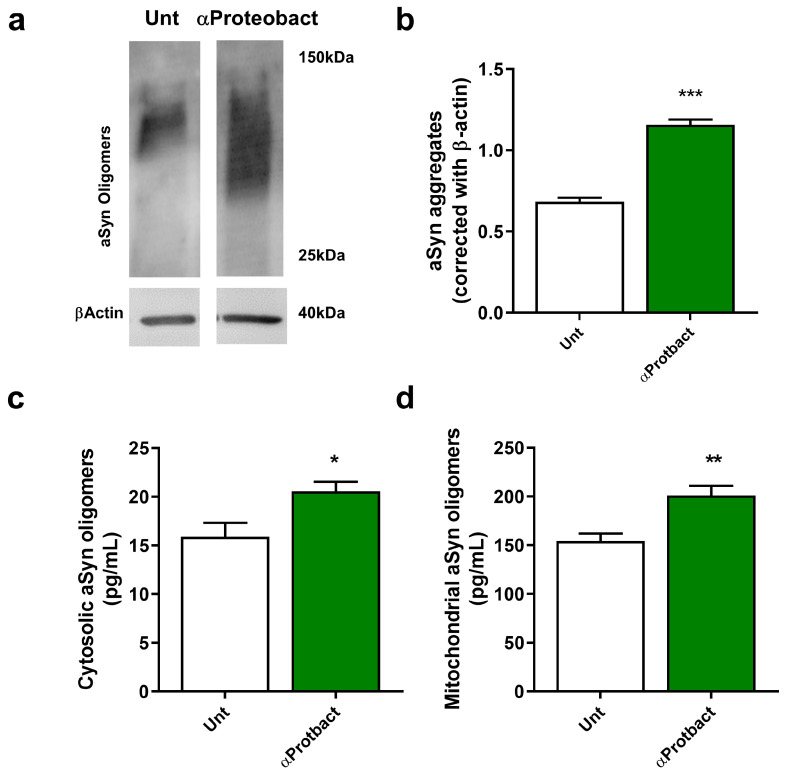
Bacterial exposure promotes α-Syn expression and aggregation. (**a**) Representative immunoblot showing α-Syn monomer and oligomers in mesencephalic neuronal cultures infected with 1 × 10^6^/mL bacteria for 48 h. The blots were re-probed for β-Actin to confirm equal protein loading. (**b**) Densitometric analyses of the levels of α-Syn normalized against β-Actin. (*n* = 3, *** *p* = 0.0003). (**c**) Cytosolic α-Syn oligomeric levels from primary mesencephalic neuronal cultures exposed to 1 × 10^6^/mL bacteria for 48 h were calculated using an ELISA kit. Values are pg/mL (*n* = 4, * *p* = 0.0346). (**d**) Mitochondrial α-Syn oligomeric levels from primary mesencephalic neuronal cultures exposed to 1 × 10^6^/mL bacteria for 48 h were calculated using an ELISA kit. Values are pg/mL (*n* = 7, ** *p* = 0.0028). Data represent mean + SEM. Statistical analysis was performed using Unpaired Student’s *t*-test.

Recently, it has been hypothesized that α-Syn expression in neurons could be part of innate immune response [11,46]. We observed that neurons exposed to the proteobacterium induced α-Syn expression and aggregation (Figure 2a,b; *n* = 3 and c; *n* = 4). Previous reports indicate that α- Syn also localizes to mitochondria and contributes to the disruption of key mitochondrial processes [47,48]. We show that α- Syn oligomers also accumulate in the mitochondria (Figure 2d; *n* = 7) after bacterial exposure.

### 2.2. Mitochondrial Dysfunction: A Positive Feedback Loop to Potentiate Innate Immunity Activation

**Figure 3:**
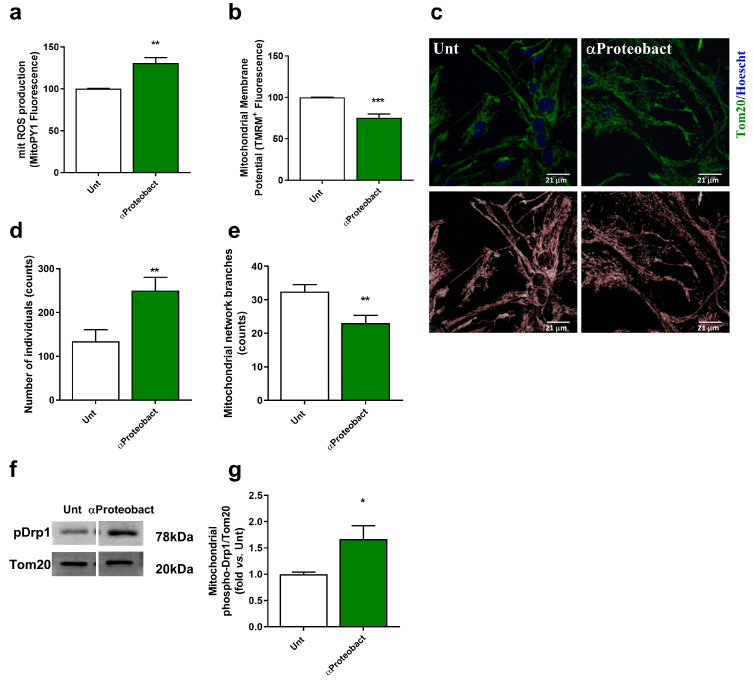
Bacteria induce neuronal mitochondrial dysfunction and fragmentation. Primary mesencephalic neuronal cultures were exposed to 1 × 10^6^/mL bacteria (α-Proteobacteria) for 48 h. (**a**) Mitochondrial ROS production was assessed using the fluorescent dye MitoPY1 (*n* = 4, ** *p* = 0.0029). (**b**) Changes in mitochondrial membrane potential (ΔΨm) were assessed using the fluorescent cationic dye TMRM (*n* = 5, *** *p* = 0.0007). (**c**) Representative images of mitochondrial network of primary mesencephalic neurons immunostained with Tom20. (**d**,**e**) Alterations in mitochondrial network were calculated with an ImageJ Macro tool (16 images from *n* = 4). (**d**) Number of mitochondrial individuals (*n* = 4, ** *p* = 0.007). (**e**) Number of mitochondrial network branches (*n* = 4, ** *p* = 0.0043). (**f**) Representative immunoblot for phospho-Drp1 levels. The blots were re-probed for TOM20 to confirm equal protein loading and mitochondrial fraction purity. (**g**) Densitometric analysis of phospho-Drp1 levels. Data are expressed relatively to Unt neurons (*n* = 4–3, * *p* = 0.0270). Scale bars = 21 µm. Data represent mean + SEM. Statistical analysis was performed using Unpaired Student’s *t*-test.

Aberrant α- Syn mitochondrial interaction has been associated with mitochondrial dysfunction, increased mitochondrial reactive oxygen species (ROS) production and mitochondrial fragmentation [49]. We observed an increase in mitochondrial ROS production in neurons exposed to the bacteria (Figure 3a; *n* = 4) and a decrease in mitochondrial membrane potential (Figure 3b; *n* = 5). Upon dysfunction, the mitochondria network fragments to allow the removal of damaged components by mitophagy [50]. We detected an increase in the number of mitochondrial individuals (Figure 3c,d; 16 images from *n* = 4) and a decrease in mitochondrial network branches (Figure 3c,e; 16 images from *n* = 4), which associates with an increase in mitochondrial levels of the phosphorylated form of the fission protein Drp1 (Figure 3f,g; *n* = 4–3). Excessive mitochondrial fission can expose DAMPs that will contribute to further activation of innate immunity, creating a positive feedback loop augmenting inflammation [7] unless they are removed by mitophagy.

**Figure 4:**
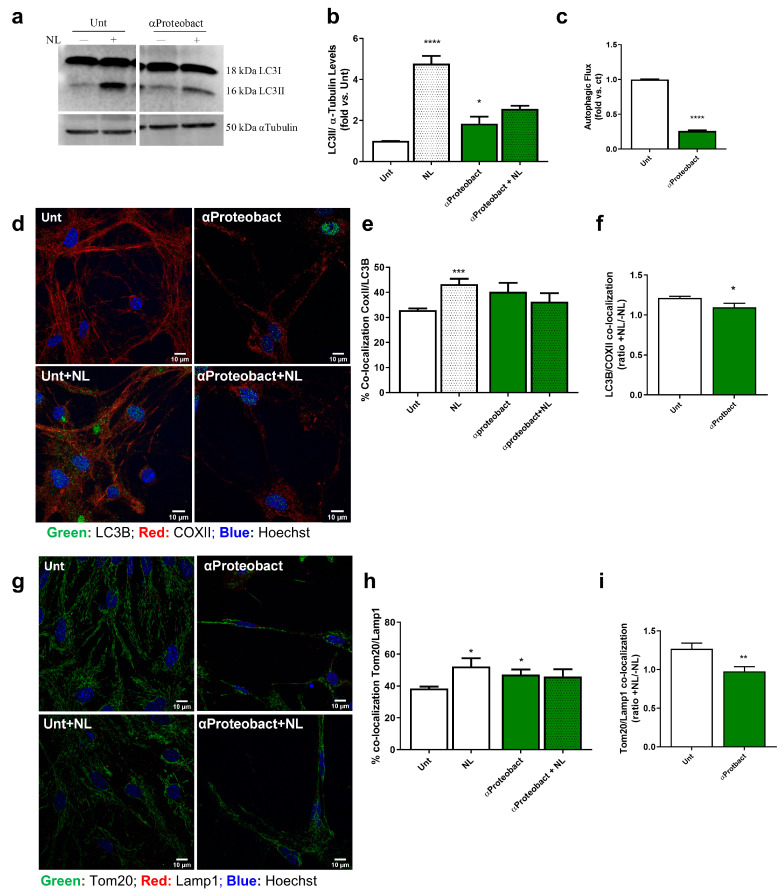
Mitophagy is differentially affected by bacterial exposure. Primary mesencephalic neuronal cultures infected with 1 × 10^6^/mL bacteria for 48 h in the presence or absence of lysosomal inhibitors (NHCl + leupeptin; NL, last 4 h) were examined by immunoblotting. (**a**) Representative immunoblot for LC3B-I and II levels. (**b**) Autophagic vacuoles basal levels (LC3-II basal densitometric values) were determined (*n* = 4, Unt vs. NL,**** *p* < 0.0001; Unt vs. αProteobact, * *p* = 0.0473). (**c**) Autophagic flux was determined (ratio of LC3-II densitometric value of NL-treated samples over the corresponding untreated samples (*n* = 4, Unt vs. αProteobact, **** *p* < 0.0001). The blots were re-probed for α-tubulin to confirm equal protein loading. (**d**) Co-localization between autophagic vacuoles (labeled in green with LC3B antibody) and mitochondria (labeled in red with COXII antibody) was visualized by immunofluorescence (*n* = 6 images). Hoechst 33342-stained nuclei are in blue. (**e**) Percentage of LC3B and COXII co-localization was calculated using Image J as described in Material and Methods (*n* = 3, Unt vs. Unt + NL, *** *p* = 0.0009). (**f**) Assessment of LC3B and COXII co-localization as the ratio between +NL/-NL treatments (*n* = 3, * *p* = 0.0487). (**g**), Co-localization between mitochondria (labeled in green with Tom20 antibody) and lysosomes (labeled in red with Lamp1 antibody) was visualized by immunofluorescence (*n* = 6–8 images). Hoechst 33342- stained nuclei are in blue. (**h**) Percentage of Tom20 and Lamp1 co-localization was calculated using Image J as described in Material and Methods. (*n* = 3, Unt vs. NL, * *p* = 0.0352; Unt vs. α-Proteobact, * *p* = 0.0427). (**i**) Assessment of Tom20 and Lamp1 co-localization as the ratio between +NL/-NL treatments (*n* = 3, ** *p* = 0.0087). Data represent mean + SEM. Scale bars = 10 µm Statistical analysis was performed using Unpaired Student’s *t*-test to compare NL treatments vs. respective control group and untreated cells versus bacterial-exposed cells.

To determine autophagy, we used NH4Cl plus leupeptin (NL) to inhibit lysosomal hydrolases and accurately determine autophagic flux. We observed that LC3II levels increased upon bacteria exposure (Figure 4a,b; *n* = 4), but its levels did not increase after intralysossomal protein degradation inhibition, which indicates a decrease in autophagic flux (Figure 4c; *n* = 4). Despite the marginal increase in the formation of mitophagosomes (Figure 4d,e; 6 images from *n* = 3) and autolysosomes (Figure 4g,h; 6–8 images from *n* = 3), we clearly see deficient signaling either in the formation of mitophagosomes (Figure 4f; *n* = 3) or their fusion within the lysosome (Figure 4i; *n* = 3), which indicates a decreased turnover of dysfunctional mitochondria.

## 3. Discussions

The goal of this study was to investigate the potential influence of bacteria–mitochondria communication on PD-related neuronal degeneration. This study reveals that an extracellular proteobacterium is capable of activating neuronal innate immunity, namely cytokine production and α-Syn expression that ultimately target the mitochondria. Alterations of neuronal mitochondria dynamics are crucial to PD neurodegenerative process, which contributes to creating a positive feedback loop to further activate innate immunity.

Proteobacteria that represent one of the most diverse bacterial phyla are gram-negative and LPS -producing bacteria [51] that have been proposed to be at the origin of mitochondria [52,53]. Plasma membrane TLR4, also expressed in neurons [54], are activated by LPS (endotoxin) to induce pro-inflammatory responses to invade pathogens [55]. This signaling pathway culminates in the activation of NF-kB that will target inflammatory genes, such as TNFα, Il-1β and IL-6, to trigger neuroinflammation and eliminate the bacterial aggressor [56]. We have exposed mesencephalic neurons to an environmental proteobacterium and observed an increase in TLR4 expression, which upon activation, induced the release of pro-inflammatory cytokines, namely IL-1β, TNFα and IL-6. Our group has previously shown, in pure cortical neurons and mesencephalic neurons, that a bacterial toxin (BMAA) activated innate immunity through TLR4 signaling [11,32]. Moreover, we observed that innate immunity activation in cortical neurons also led to an increased expression and aggregation of Abeta peptide [32] and that mesencephalic neurons innate immunity activation was correlated with an increased expression and aggregation of α-Syn [11]. Using a proteobacterium strain as a challenger, our data clearly show an increase in α-Syn aggregation, which corroborates the potential key role of α-Syn in neuronal innate immunity responses. The activation of TLRs in non-immune cells such as neurons has a pivotal role in recognizing exogenous and endogenous stimuli to trigger inflammatory responses that, in the short run, might have protective effects, namely to clear protein oligomers such as α-Syn in PD, delaying disease progression [56]. Indeed, an increase of pro-inflammatory markers in the blood [57,58], brain parenchyma [59,60] and cerebrospinal fluid [61] in PD patients and patients with other Synucleinopathies was observed, which indicates a chronic activation of TLRs and neuroinflammation that may lead to neurodegeneration. Additionally, it is believed that α-Syn misfolding and mitochondrial dysfunction may trigger neuroinflammation associated with PD [62]. The role of α-Syn is not yet completely understood, but upon expression, it may oligomerize and translocate into the mitochondria, where it interferes with mitochondrial respiration [63]. Indeed, mitochondrial dysfunction induced by α-Syn has been demonstrated [47]. Moreover, in PD patients’ substantia nigra, accumulation of α-Syn oligomers was correlated with mitochondrial complex I deficiency [64]. Further corroborating this data, we show that α-Syn enters the mitochondria after neuronal innate immunity activation by a bacterial strain and induces its dysfunction. Relevant data also revealed that other stressors, namely the bacteria toxin BMAA, also potentiate the accumulation of α-Syn in the mitochondria and are associated with its dysfunction and network fragmentation [11]. One prominent consequence of mitochondrial dysfunction is the induction of its fragmentation in order to eliminate the defective parts of the network by mitophagy and keep cellular homeostasis [65]. Mitochondrial dysfunction and consequent fragmentation will expose DAMPs that initiate multiple inflammatory pathways [62]. Cells developed efficient mechanisms to prevent auto-inflammatory or auto-immune responses towards mitochondrial DAMPs, establishing a state of immune tolerance towards mitochondria [53]. Indeed, an efficient mitophagy allows the removal of defective mitochondria [50] but also plays a role in NLRP3 inflammasome pathway [52]. The activation of NLRP3 inflammasome requires two signals, the first related to TLR activation and production of pro-IL1β, and the second signal is based on the detection of mitochondrial DAMPs that leads to the activation of caspase-1 that cleaves the pro-IL1β, thus allowing the release of these inflammatory cytokines in the extracellular milieu [52]. Our data show that despite the fragmentation of the mitochondrial network after bacteria-induced innate immunity activation, mitophagy is not functioning properly to avoid further activation of NLRP3 inflammasome. Previous data in PD models clearly show that mitochondrial dysfunction impairs mitophagy due to altered microtubule-dependent traffic [13,42,66]. Chung and coworkers showed that neuronal activation of TLR4 by activated microglia led to neuronal autophagy impairment and α-Syn aggregate accumulation [67]. Nevertheless, it was previously demonstrated that neuronal α-Syn may be released to activate microglia. Activated microglia will then degrade α-Syn by selective autophagy via TLR4 activation, which induces transcriptional upregulation of p62/SQSTM1 through the NF-κB signaling pathway [68]. Interestingly, recent data show that mitochondria and α-Syn may be transferred between microglia cells [69], lowering dysfunctional mitochondria and α-Syn burden, thus attenuating the inflammatory profile. Although we do not test this hypothesis, we postulate that neurons may be initially involved in the neuroinflammation signaling pathway by releasing dysfunctional mitochondria and α-Syn aggregates, eventually through extracellular vesicles, to activate microglia cells. This initial activation of microglia might have protective effects regarding the clearance of α-Syn, thus delaying disease progression while chronic activation will lead to neurodegeneration.

Neuronal responses after exposure to proteobacteria clearly show a close interconnection between innate immunity activation and mitochondrial dysfunction [70], which allows us to consider the key role of microbes in PD development. Recently, the existence of a BrainBiota that may play a role in brain development and immunity was proposed [71]. It was postulated that low-level bacteria would travel through the gut–brain axis and colonize the brain during fetal development. Later in life, and upon BBB leakage, a characteristic mark of PD, bacteria would reach the brain, change the BrainBiota and contribute to chronic inflammation [71]. Our findings tend to support the hypothesis that translocation of PAMPs (bacteria metabolites, bacterial vesicles or even bacteria) resulting from a dysbiotic leaky gut in “gut-first” PD cases and their access to neurons of the central nervous system may affect neuronal function through mitochondrial signaling and eventually trigger cellular processes characteristic of PD neuropathology.

## 4. Materials and Methods

Materials are depicted in Table S1 and Experimental Flowchart in Figure S1 in Supplementary Material.

### 4.1. Primary Mesencephalic Cultures Preparation and Treatments

Primary mesencephalic neuronal cultures were performed by harvesting the mesencephalon of C57Bl/6 mice embryos brains at gestation day 14/15 and cultured as described previously [72]. Embryos were collected in Hanks’ balanced salt solution (HBSS) [5.36 mM KCl, 0.44 mM KH_2_PO_4_, 137 mM NaCl, 4.16 mM NaHCO_3_, 0.34 mM NaH_2_PO_4_.H_2_O, 5 mM glucose, 5.36 mM sodium pyruvate, 5.36 mM Hepes, 0.001% Fenol Red, (pH 7.2)] under aseptic conditions. Brains were dissected and the mesencephala were carefully harvested and submerged in HBSS. Collected mesencephala were trypsinized (0.5 g/L) for 10 min at 37 °C. Trypsin action was halted with the addition of trypsin inhibitor (type II-S; 0.75 g/L) in HBSS containing DNase I (0.04 g/L), followed by mechanical dissociation. Cells were then centrifugated at 1000 rpm for 5 min at 4 °C. The pellet was resuspended and washed in HBSS and centrifugated again at 1000 rpm for 5 min at 4 °C. The pellet was then suspended in fresh Neurobasal medium supplemented with 2 mM L-glutamine, 2% B-27 supplement, penicillin (100,000 U/L) and streptomycin (100 mg/L) and 1% heat-inactivated FBS and seeded on poly-L-lysine (0.1 g/L)-coated dishes at a density of 1.3 × 10^6^ cells/mL. For mitochondrial membrane potential and mitochondrial ROS production experiments, neurons were seeded on poly-L lysine (0.1 mg/mL)-coated 24-well plates at a density of 1.3 × 10^6^ cells/mL. Cultures were grown at 37 °C in a fully humidified air atmosphere containing 5% CO_2_. Half of the medium was changed every other day to serum-free and antibiotic-free medium. At DIV3, cultures were treated with 1:2000 5-Fluoro-2′-deoxyuridine (FDU) to inhibit glial cell proliferation. For autophagy experiments, 20 mM NH4Cl and/or 20 μM Leupeptin (Sigma, St. Louis, MO, USA) were added to the culture medium 4h prior to protein extracts preparation. NH4Cl in combination with Leupeptin allows for the blockage of several types of autophagy by increasing lysosomal lumen pH. This increase halts the activity of lysosomal proteases activity, maintaining the activity of the intracellular proteolysis systems [73].

### 4.2. Bacterial Strain, Culture Conditions and Treatment

The alphaproteobacterium strain used in this study belongs to the species Labrys neptuniae, as confirmed from the 16s rRNA gene sequence (Figure S2 in Supplementary Material) amplified with primers 27f and 1492R (Table S1) and sequenced (Eurofins). The strain was streaked on a Tryptic Soy agar (TSA) plate and grown overnight at 35 °C. Bacterial biomass (a loopful of cells) was then suspended in sterile PBS to a final OD600nm = 0.1 and administered to the neuronal cultures at a MOI = 10 for 48 h at 37 °C in a humidified chamber with a 95% air/5% CO_2_ atmosphere. For all experimental procedures, controls were performed in the absence of bacteria. 

### 4.3. Cellular Extracts Preparation

Protein extracts were prepared for western blotting and assessment of innate immunity pathway markers by ELISA as described in [11]. Mesencephalic neurons were washed with PBS 1× and lysed in 1% Triton X-100 containing hypotonic lysis buffer (25 mM HEPES, 2 mM MgCl2, 1 mM EDTA and 1 mM EGTA, pH 7.5 supplemented with 2 mM sodium orthovanadate, 50 mM of sodium fluoride, 2 mM DTT, 0.1 mM PMSF and a 1:1000 dilution of a protease inhibitor cocktail from Sigma (St. Louis, MO, USA). Scrapped cellular suspensions were frozen three times in liquid nitrogen and centrifuged at 20,000× *g* for 10 min. The supernatants were collected and stored at −80 °C until further use.

Mitochondrial fractions were prepared for α-Syn determination using an ELISA kit. To this end, cell cultures were washed in PBS 1× and scraped in a buffer containing 250 mM sucrose, 20 mM Hepes, 1 mM EDTA, 1 mM EGTA, supplemented with 2 mM sodium orthovanadate, 50 mM of sodium fluoride, 0.1 mM PMSF, 2 mM DTT and 1:1000 dilution of a protease inhibitor cocktail followed by manual homogenization. Cell suspensions were then centrifuged at 492× *g* for 12 min at 4 °C and the resulting supernatant was centrifuged again at 11,431× *g* for 20 min at 4 °C. The resulting pellet corresponding to the mitochondrial fraction was resuspended in buffer solution and frozen three times in liquid nitrogen.

To analyze innate immunity markers with Elisa kits, cytosolic fractions were prepared by washing neuronal cultures with PBS 1× and lysing cells with lysis buffer (10 mM HEPES; 3 mM MgCl_2_; 1 mM EGTA; 10 mM NaCl, pH 7.5, supplemented with 2 mM DTT, 0.1 mM PMSF and a 1:1000 dilution of a protease inhibitor cocktail) supplemented with 0.1% Triton X-100. After scraping neurons, the suspensions were incubated on ice for 40 min and then centrifuged at 2300× *g* for 10 min at 4 °C. The supernatant corresponding to the cytosolic fraction was stored at −80 °C until further use. Protein content was assessed by using Pierce™ BCA Protein Assay Kit (Thermo Scientific, Rockford, IL, USA) according to the manufacturer’s instructions.

### 4.4. Western Blotting

Western blotting was performed as previously described in [13]. Samples were diluted in 6× sample buffer (4× Tris-Cl/SDS, pH 6.8, 30% glycerol, 10% SDS, 0.6 M DTT, 0.012% bromophenol blue) and boiled at 95 °C for 5 min. For α-Syn oligomers determination, samples were suspended in 2× PAGE sample buffer (40% glycerol, 2% SDS, 0.2 M Tris-HCl pH 6.8, 0.005% Coomassie Blue) and loaded under non-denaturing conditions. Ran gels were transferred onto PVDF membranes (Millipore, Billerica, MA, USA) and blocked for 1 h with 3% BSA, 0.1% Tween in Tris-buffered solution (TBS) at RT. Primary antibodies were incubated overnight at 4 °C with gentle shaking: 1:100 anti-TLR4 from Santa Cruz Biotechnology (Santa Cruz, CA, USA), 1:100 monoclonal anti-α- Syn LB509 from Zymed Laboratories Inc. (South San Francisco, CA, USA); 1:1000 anti-phospho-Drp1 from Cell Signaling (Danvers, MA, USA); 1:1000 polyclonal anti-Tom20 from Santa Cruz Biotechnology (Santa Cruz, CA, USA) and 1:1000 polyclonal anti-LC3B from Cell Signaling (Danvers, MA, USA). 1:10,000 monoclonal anti-α-tubulin from Sigma (St. Louis, MO, USA), 1:5000 β-tubulin from Sigma (St. Louis, MO, USA) or 1:5000 β-actin from Sigma (St. Louis, MO, USA) were used to normalize band intensities.

After primary incubation, membranes were washed with TBS-T three times for 5 min each and then incubated with the appropriate secondary antibody for 2 h at RT. After three washes with TBS-T, the chemical fluorescence of bands was enhanced with chemical fluorescence reagent (ECF from GE Healthcare, Piscataway, NJ, USA). Membranes were revealed using a Bio-Rad Chemidoc System. Western blot band densities were assessed using Quantity One Software (Bio-Rad).

Band intensities were normalized to housekeeping genes (β-actin and α-tubulin were used as cytosolic samples loading control and TOM20 for mitochondrial samples) and relative densities were calculated against control conditions for each membrane.

### 4.5. Immunocytochemistry and Confocal Microscopy Analysis

For immunocytochemistry experiments, mesencephalic neurons were grown in Ibidi 8-well µ-Slides as described in [11]. Following bacteria exposure, cultures were washed twice with PBS 1× and fixed with 4% paraformaldehyde for 20 min at RT. After fixation, cells were washed twice in PBS 1× for 5 min each and permeabilized with 0.2% Triton X-100 in PBS for 20 min at RT. After three washes with PBS 1× for 5 min each, unspecific binding was blocked with a 10% goat serum solution for 1 h at 37 °C. Primary antibodies were then incubated overnight at 4 °C in a 1% goat serum solution: 1:100 polyclonal anti-Tom20 from Santa Cruz Biotechnology (Santa Cruz, CA, USA); 1:400 rabbit monoclonal anti-LC3 XP^®^ from Cell Signaling (Danvers, MA, USA); 1:200 anti-SDHA from Abcam (Cambridge, UK) and 1:100 anti-Lamp1 (clone H4A3) from the Developmental Studies Hybridoma Bank (University of Iowa, Iowa City, IA, USA). After, cells were incubated with the respective secondary antibodies: 1:250 Alexa Fluor 488 and 1:250 Alexa Fluor 594 from Molecular Probes (Eugene, OR, USA). After three washes with PBS 1× for 5 min each, cells were incubated with Hoechst 15 μg/μL for 15 min at RT. After two washes with PBS 1× for 5 min each, 4-88 Mowiol (Sigma; St. Louis, MO, USA) mounting medium was applied to the wells.

Images were obtained on a Zeiss LSM 710 confocal workstation (Zeiss Microscopy, Germany) using a Plan-Apochromat/1.4NA 63 lens. Tom20/Lamp1 and LC3/SDHA co-localizations were evaluated using the JACoP plug-in of the ImageJ software as previously described [11]. First, threshold of images was obtained to improve image quality, and mitochondrial footprint was calculated. Then, mitochondrial networks were skeletonized to calculate the remaining parameters. For each condition, a minimum of 20 cells were examined.

### 4.6. Evaluation of Mitochondrial Membrane Potential (Δψm)

Mitochondrial membrane potential (ΔΨmit) was evaluated by using the tetramethylrhodamine methyl ester dye (TMRM) (Molecular Probes, Eugene, OR, USA) [74]. This dye enters cells through diffusion and accumulates essentially in the mitochondria due to its negatively charged lumen. As TMRM is positively charged, functional mitochondria are able to retain this probe. Thus, a decrease in TMRM retention associates with a decreased Δψm. After treatments, neuronal cultures with PBS 1× and loaded with 300 nM TMRM in Krebs buffer (132 mM NaCl, 4 mM KCl, 1.4 mM MgCl_2_, 6 mM glucose, 10 mM HEPES, 10 mM NaHCO_3_ and 1 mM CaCl_2_, pH = 7.4) for 2 hr at 37 °C in a humidified chamber and protected from light. Basal readings were recorded for the first 5 min at 37 °C (λex = 540 nm and λem = 590 nm). 1 μM FCCP (protonophore) and 2 μg/mL oligomycin (inhibitor of H^+^ transporting ATP synthase and an inhibitor of Na^+^/K^+^ transporting ATPase) were then added to each well to allow for maximal mitochondrial depolarization and to prevent ATP synthase reversal, respectively. Readings were performed for another 3 min at 37 °C. TMRM retention ability determined as the difference between the total fluorescence (after depolarization) and the basal value of fluorescence. Results were expressed as a percentage of the dye retained within the untreated WT neurons. Measurements were performed using a Spectramax Plus 384 spectrofluorometer (Molecular Devices, Sunnyvale, CA, USA).

### 4.7. Determination of Mitochondrial-Derived Reactive Oxygen Species

MitoPy1 is a fluorescent probe that measures the concentration of hydrogen peroxide (H_2_O_2_) in mitochondria. After treatments, neuronal cultures were incubated with 300 nM of MitoPY1 dye for 1h in Krebs medium at 37 °C as described in [32]. Basal fluorescence readings were performed for 5 min (λexc = 503 nm; λem = 540 nm). Neurons were then incubated with 5 µM of rotenone (complex I inhibitor) to determine mitochondrial vulnerability, and measurements were taken for the following 30 min (λexc = 503 nm; λem = 540 nm). Amplitudes were obtained by subtracting basal readings from peak values under rotenone challenge and were expressed in relative values to untreated neurons.

### 4.8. Caspase-1 Activity Assay

Caspase.1 activation was assessed as described in [32]. Briefly, 40 μg of protein extracts were incubated in a reaction buffer (25 mM HEPES pH 7.5, 0.1% (*w*/*v*) 3[(3-cholamidopropyl)dimethylammonio]-propanesulfonic acid (CHAPS), 10% (*w*/*v*) sucrose, 2 mM DTT) with 100 μM of the colorimetric substrate for caspase-1 from Sigma Chemical Co. (St. Louis, MO, USA) for 2 h at 37 °C. Reaction extent was measured at 405 nm using a Spectramax Plus 384 spectrophotometer (Molecular Devices, Sunnyvale, CA, USA).

### 4.9. Inflammatory Markers and α-Syn Oligomers Determination by ELISA

To determine the cytokine levels in neuronal extracts, 25 μg of protein were used for each ELISA kit. NFκB p65, IL-1β, TNF-α, IL-6 and α-Syn oligomers ELISA kits were used per the manufacturer’s instructions as described in [11]. Absorbance was assessed at 450 nm in a SpectraMax Plus 384 multiplate reader. Results were expressed as pg/mL for all markers except results for NFκB, which were expressed as μg/mL protein.

### 4.10. Statistical Analysis

All the results were obtained from at least 3 independent experiments done in duplicates. All data are represented as the mean ± SEM. Normality distribution analysis (Shapiro-Wilk’s test) was applied to choose the subsequent parametric or non-parametric tests. Unpaired Student’s *t*-test was used, and significant values are shown as: * *p* < 0.05, ** *p* < 0.01, *** *p* < 0.001, **** *p* < 0.0001.

## Data Availability

The datasets generated during and/or analyzed during the current study are not publicly available since we do not have a repository, but they are available from the corresponding author on reasonable request.

## Supplementary Materials

The supporting information can be downloaded at: https://www.mdpi.com/article/10.3390/ijms24054339/s1.
