# Supplementary material for: The Role of Bacteria–Mitochondria Communication in the Activation of Neuronal Innate Immunity: Implications to Parkinson’s Disease
Source: Int J Mol Sci. 2023 Feb 22;24(5):4339. doi: 10.3390/ijms24054339 (PMC10001700; doi:10.3390/ijms24054339)
Supplement: Supplementary file 1 [file ijms-24-04339-s001.zip › ijms-2130308-supplementary.pdf]

Table S1. List of Reagents

REAGENT or RESOURCE	SOURCE
**Antibodies – Western Blotting**	
Mouse anti-TLR4	Santa Cruz Biotechnology (Cat. No. sc-293072)
Mouse anti-α-synuclein LB509	Zymed Laboratories Inc. (Cat. No. 180215)
Rabbit anti-phospho DRP1 (serine 616)	Cell Signaling (Cat. No. 3455s)
Rabbit anti-TOM20	Santa Cruz Biotechnology (Cat. No. sc-11415)
Rabbit anti-LC3B	Cell Signaling (Cat. No. 3868)
Mouse anti-α-tubulin	Sigma (Cat. No. T6199)
Mouse anti-β-tubulin	Sigma (Cat. No. T7816)
Mouse β-actin	Sigma (Cat. No. A5441)
Goat anti-mouse IgG	Thermo Fisher Scientific (Cat. No. 31320)
Goat anti-rabbit IgG	GE Healthcare (Cat. No. NIF1317)
**Antibodies – IF**	
Mouse Anti-SDHA	Abcam (Cat. No. ab14715)
Mouse anti-Lamp1	clone H4A3 from the Developmental Studies Hybridoma Bank
Goat anti-mouse Alexa Fluor 488	Molecular Probes, Life Technologies (Cat. No. A11001)
Goat anti-mouse Alexa Fluor 594	Molecular Probes, Life Technologies (Cat. No. A11005)
Goat anti-rabbit Alexa Fluor 488	Molecular Probes, Life Technologies (Cat. No. A11008)
**Kits**	
NFκB p65 Total SimpleStep ELISA Kit	Abcam (Cat. No. ab176648)
Mouse IL-1β Quantikine ELISA	MyBioSource (Cat. No. MBS724099)
Mouse TNF-α Quantikine ELISA	R&D Systems (Cat. No. MTA00B)

Mouse IL-6 Quantikine ELISA	R&D Systems (Cat. No.M6000D)
α Synuclein oligomer (SNCO α) ELISA Kit	R&D Systems (Cat. No. MLB00C)
**Chemicals**	
Ammonium chloride (NH_4Cl)	Merck KGaA (Cat. No. 9434)
Tetramethylrhodamine, Methyl Ester, Perchlorate (TMRM)	Molecular Probes, Life Technologies (Cat. No. T668)
Caspase 1 substrate	Sigma (Cat. No. SCP0066)
Hoechst 33342	Invitrogen (Cat. No. H1399)
Leupeptin	Sigma (Cat. No. L2023)
**Primers**	
27F (5'-AGAGTTTGATCMTGGCTCAG)	Eurofins Genomics Italy
1525R (5′–AGAAAGGAGGTGATCCAGCC)	Eurofins Genomics Italy

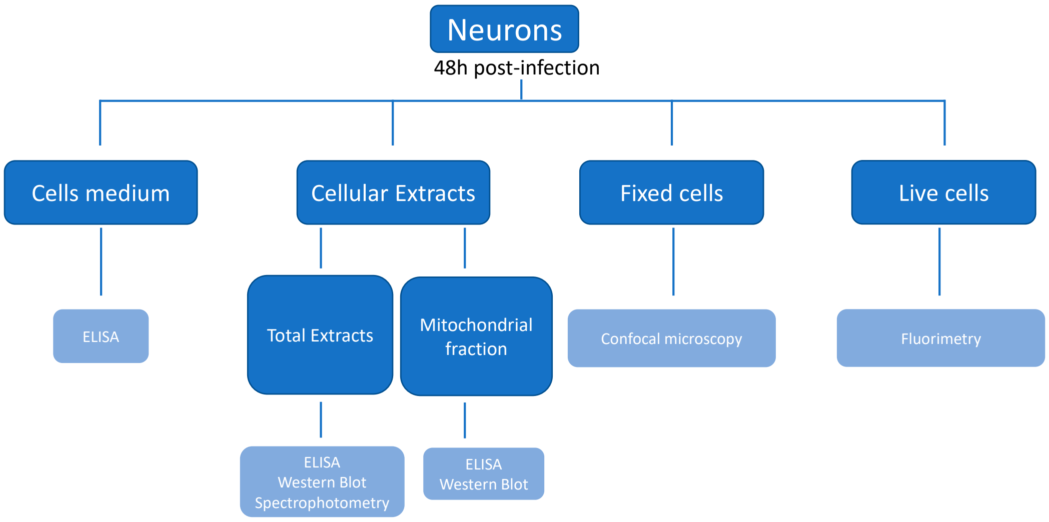

Figure S1. Experimental Flowchart

>16s rRNA (1354 nt)
TGCAAGTCGAACGCATCCTTCGGGGTGAGTGGCAGACGGGTGAGTAACGCGTGGGGATGTGCCTTGAGGTGGGGAATAACTGTGGGAAACTACAGCTAATAC
CGCATACGCCCTTTTGGGGAAAGATTTATCGCCTTTAGAGCAACCCGCGTCAGATTAGCTAGTTGGTAGGGTAATGGCCTACCAAGGCGACGATCTGTAGCT
GGTCTGAGAGGATGACCAGCCACACTGGGACTGAGACACGGCCCAGACTCCTACGGGAGGCAGCAGTGGGGAATATTGGACAATGGGCGCAAGCCTGATCCA
GCCATGCCGCGTGAGTGATGACGGCCTTAGGGTTGTAAAGCTCTTTTAACAGGGACGATAATGACGGTACCTGTAGAATAAGCCCCGGCAAACTTCGTGCCA
GCAGCCGCGGTAATACGAAGGGGGCTAGCGTTGTTCGGAATTACTGGGCGTAAAGCGCACGTAGGCGGATTGTTAAGTCGGGGGTGAAATCCTGAGGCTCAA
CCTCAGAACTGCCTTCGATACTGGCGATCTTGAGTTCGGAAGAGGTTGGTGGAACAGCTAGTGTAGAGGTGAAATTCGTAGATATTAGCTAGAACACCAGTG
GCGAAGGCGGCCAACTGGTCCGATACTGACGCTGAGGTGCGAAAGCGTGGGGAGCAAACAGGATTAGATACCCTGGTAGTCCACGCCGTAAACGATGAATGC
CAGCCGTCGGGGAGCTTGCTCTTCGGTGGCGCAGCTAACGCTTTAAGCATTCCGCCTGGGGAGTACGGTCGCAAGATTAAAACTCAGAGGAATTGACGGGGG
CCCGCACAAGCGGTGGAGCATGTGGTTTAATTCGAAGCAACGCGCAGAACCTTACCAGCCCTTGACATCCCGGTCGCGGATCACAGAGATGAGATCCTTCAG
TTCGGCTGGACCGGAGACAGGTGCTGCATGGCTGTCGTCAGCTCGTGTCGTGAGATGTTGGGTTAAGTCCCGCAACGAGCGCAACCCTCGCCCCTAGTTGCC
AGCATTAAGTTGGGCACTCTAGGGGGACTGCCGGTGATAAGCCGCGAGGAAGGTGGGGATGACGTCAAGTCCTCATGGCCCTTACGGGCTGGGCTACACACG
TGCTACAATGGCGGTGACAGTGGGAAGCGAAGGGGTGACCCCTAGCAAATCTCCAAAAGCCGTCTCAGTTCAGATTGCACTCTGCAACTCGAGTGCATGAAG
GTGGAATCGCTAGTAATCGCAGATCAGCATGCTGCGGTGAATACGTTCCCGGGCCTTGTACACACCGCCCGTCACACCATGGGAGTTGGTTTACCCGAAGGC
GCTGCGCCAACCGCAAGGAGGCAGGCGA

Figure S2. Sequence of 16S gene of *Labrys neptuniae*
